# Outcomes and costs of implementing a community-based intervention for hypertension in an urban slum in Kenya

**DOI:** 10.2471/BLT.15.156513

**Published:** 2016-04-26

**Authors:** Samuel Oji Oti, Steven van de Vijver, Gabriela B Gomez, Charles Agyemang, Thaddaeus Egondi, Catherine Kyobutungi, Karien Stronks

**Affiliations:** a African Population and Health Research Center, PO Box 10787-00100, Nairobi, Kenya.; b Amsterdam Institute for Global Health and Development, Amsterdam, Netherlands.; c Department of Public Health, University of Amsterdam, Amsterdam, Netherlands.

## Abstract

**Objective:**

To describe the processes, outcomes and costs of implementing a multi-component, community-based intervention for hypertension among adults aged > 35 years in a large slum in Nairobi, Kenya.

**Methods:**

The intervention in 2012–2013 was based on four components: awareness-raising; improved access to screening; standardized clinical management of hypertension; and long-term retention in care. Using multiple sources of data, including administrative records and surveys, we described the inputs and outputs of each intervention activity and estimated the outcomes of each component and the impact of the intervention. We also estimated the costs associated with implementation, using a top-down costing approach.

**Findings:**

The intervention reached 60% of the target population (4049/6780 people), at a cost of 17 United States dollars (US$) per person screened and provided access to treatment for 68% (660/976) of people referred, at a cost of US$ 123 per person with hypertension who attended the clinic. Of the 660 people who attended the clinic, 27% (178) were retained in care, at a cost of US$ 194 per person retained; and of those patients, 33% (58/178) achieved blood pressure control. The total intervention cost per patient with blood pressure controlled was US$ 3205.

**Conclusion:**

With moderate implementation costs, it was possible to achieve hypertension awareness and treatment levels comparable to those in high-income settings. However, retention in care and blood pressure control were challenges in this slum setting. For patients, the costs and lack of time or forgetfulness were barriers to retention in care.

## Résumé

**Objectif:**

Décrire les processus, les résultats et les coûts de la mise en œuvre d'une intervention communautaire à composantes multiples contre l'hypertension auprès d'adultes de 35 ans et plus, dans un grand bidonville de Nairobi, au Kenya.

**Méthodes:**

Cette intervention, menée en 2012–2013, s'est articulée autour de quatre composantes: sensibilisation, amélioration de l'accès au dépistage, prise en charge clinique standardisée de l'hypertension; et rétention dans les soins sur le long terme. À partir de plusieurs sources de données, notamment des registres administratifs et des enquêtes, nous avons décrit les intrants et extrants de chaque activité constitutive de cette intervention et avons évalué les résultats de chaque composante ainsi que l'impact général de l'intervention. Nous avons également estimé les coûts associés à la mise en œuvre, en utilisant une approche de chiffrage descendante.

**Résultats:**

Cette intervention a touché 60% de la population ciblée (4 049/6 780 personnes), pour un coût de 17 dollars des États-Unis ($US) par personne dépistée, et a permis le traitement de 68% (660/976) des personnes orientées vers une clinique, pour un coût de 123 $US par personne hypertendue s'étant rendue à l'une des cliniques. Sur les 660 personnes s'étant rendues à une clinique, 27% (178) ont été maintenues dans le continuum de soins, pour un coût de 194 $US par personne, et parmi ces patients, 33% (58/178) ont réussi à réguler leur pression artérielle. Le coût total de l'intervention par patient ayant réussi à réguler sa pression artérielle a été estimé à 3 205 $US.

**Conclusion:**

Avec des coûts de mise en œuvre modérés, il a été possible d'atteindre des niveaux de sensibilisation et de traitement comparables à ceux obtenus dans les pays à revenu élevé. Cependant, la rétention dans le continuum de soins et la régulation de la pression artérielle ont constitué de réels défis dans le contexte de ce bidonville. Pour les patients, les coûts, le manque de temps et l'oubli des rendez-vous médicaux ont été des freins pour poursuivre le continuum de soins.

## Resumen

**Objetivo:**

Describir los procesos, resultados y costes de la implementación de una intervención de varios componentes basada en la comunidad para la hipertensión en adultos de > 35 años en un gran barrio pobre de Nairobi, Kenya.

**Métodos:**

La intervención realizada en 2012-2013 se basaba en cuatro elementos: toma de conciencia; acceso mejorado a revisiones; gestión clínica de la hipertensión estandarizada; y recepción de atención a largo plazo. Utilizando numerosas fuentes de datos, incluidos expedientes administrativos y encuestas, se describieron las contribuciones y el rendimiento de cada actividad de intervención y se calcularon los resultados de todos los componentes, así como el impacto de la intervención. También se calcularon los costes relacionados con la implementación utilizando un enfoque de gastos descendente.

**Resultados:**

La intervención llegó al 60% de la población objetivo (4 049/6 780 personas), con un coste de 17 dólares estadounidenses (USD) por persona examinada y un acceso al tratamiento del 68% (660/976) de las personas, con un coste de 123 USD por persona con hipertensión que acudió a la clínica. De las 660 que acudieron a la clínica, el 27% (178) recibieron atención, con un coste de 194 USD por persona atendida; y de dichos pacientes, el 33% (58/178) lograron controlar la tensión arterial. El coste total de la intervención por paciente con tensión arterial controlada fue de 3 205 USD.

**Conclusión:**

Con costes moderados de implementación, fue posible lograr una toma de conciencia sobre la hipertensión y niveles de tratamiento comparables con aquellos de lugares con ingresos altos. No obstante, la recepción de atención y el control de la tensión arterial fueron muy complicados en este barrio pobre. Para los pacientes, el coste y la falta de tiempo o el olvido fueron obstáculos para recibir atención médica.

## ملخص

**الغرض:**

وصف العمليات والحصائل والتكاليف الناتجة عن تنفيذ تدخل مجتمعي متعدد المكونات لارتفاع ضغط الدم في أوساط البالغين الذين تزيد أعمارهم عن 35 **  **عامًا في أحد الأحياء الحضرية الفقيرة الكبيرة في نيروبي، كينيا.

**الطريقة:**

كان التدخل المقام في الفترة من عام 2012 إلى عام 2013 قائمًا على أربعة مكونات: زيادة مستوى التوعية؛ وتحسين سبل الخضوع إلى الفحص؛ والمعالجة السريرية الموحدة لارتفاع ضغط الدم؛ والاستمرار في تلقي الرعاية على المدى الطويل. قمنا بوصف المدخلات والنواتج لكل نشاط من أنشطة التدخل وتقدير الحصائل الناتجة لكل مكون وتأثير التدخل وذلك باستخدام مصادر متعددة من البيانات، بما في ذلك السجلات والمسوح الإدارية. كما قمنا أيضًا بتقدير التكاليف المرتبطة بعملية التنفيذ، وذلك باتباع نهج حساب التكاليف من المستويات العليا إلى المستويات الدنيا.

**النتائج:**

بلغت نسبة التدخل 60‏% من السكان المستهدفين (4049/6780 من الأشخاص)، بتكلفة 17 دولارًا أمريكيًا للشخص الواحد الذي خضع للفحص، وتوفير إمكانية الحصول على العلاج بنسبة ‏68% (660/976) من الأشخاص المحالين للعلاج بتكلفة 123 **  **دولارًا أمريكيًا للشخص الواحد الذي يعاني من ارتفاع ضغط الدم والذين ذهبوا لتقي العلاج في العيادة. من بين إجمالي الأشخاص الذين حضروا إلى العيادة والبالغ عددهم 660 شخصًا، كان ‏‏27% (178) منهم مستمرون في تلقي الرعاية، بتكلفة قدرها 194 دولارًا أمريكيًا لكل شخص مستمر في تلقي الرعاية؛ ومن بين هؤلاء المرضى، استطاع ‏33‏% (58/178) منهم السيطرة على ضغط الدم. وبلغت التكلفة الإجمالية للتدخل الخاص بكل مريض يتمتع بضغط دم مضبوط 3205 دولارات أمريكية.

**الاستنتاج:**

كان من الممكن تحقيق التوعية بخطورة ارتفاع ضغط الدم وتنفيذ مستويات العلاج المماثلة لتلك الإمكانيات الموجودة في الأماكن ذات الدخل المرتفع من خلال تكاليف التنفيذ المعتدلة. إلا أن الاستمرار في تلقي الرعاية والسيطرة على ضغط الدم كانت من الأمور التي تمثل تحديًا في هذا الحي الحضري الفقير. فبالنسبة للمرضى، كانت التكاليف أو ضيق الوقت أو النسيان هي الأمور التي تمثل عقبات نحو الاستمرار في تلقي الرعاية.

## 摘要

**目标:**

旨在对一项基于社区的多组分干预的过程、结果以及成本进行描述，该干预针对居住在肯尼亚内罗毕一个大型贫民窟的 35 岁以上成年人患有的高血压疾病开展。

**方法:**

2012-2013 进行的干预基于 4 个不同组分： 增强意识、提升筛查普及度、标准化高血压临床管理、以及实现长期留院护理。 通过使用多来源数据，包括行政记录和调查，我们描述了每项干预活动的输入和输出，并且预测了每个组份的结果以及干预影响。我们还通过使用自上而下的成本计算方法预测了执行相关成本。

**结果:**

该干预范围涵盖了 60% 的目标人群（4049/6780 人），平均筛查成本为每人 17 美元 (US$)，并且为 68% (660/976) 的参与人员提供了治疗，成本为每位就诊的高血压患者 123 美元 (US$)。在就诊的 660 人中，27% (178) 人留作住院护理，平均成本为每人 194 美元 (US$)；而上述患者中，33% (58/178) 患者的血压得以控制。总体干预成本为每位血压得以控制的高血压患者 3205 美元 (US$)。

**结论:**

有了适当的干预成本，实现与高收入环境可比拟的高血压防治意识以及治疗水平是可行的。 然而，在贫民窟环境中，留院护理以及血压控制均提出很大挑战。 对于患者来说，成本以及时间不足或者健忘都是阻碍留院护理的障碍。

## Резюме

**Цель:**

Описать ход и результаты проведения комплексного общественного вмешательства, нацеленного на борьбу с гипертонией среди взрослого населения в возрасте старше 35 лет в крупных трущобах г. Найроби, Кения, и затраты, связанные с ним.

**Методы:**

Вмешательство, проводимое в 2012–2013 гг., базировалось на четырех составляющих: просветительской работе, повышении доступности скринингового обследования, стандартизированном клиническом лечении гипертонии и долгосрочном удержании пациентов в сфере оказания помощи. С помощью многочисленных источников данных, в том числе административных записей и опросов, авторы статьи описали затраченные ресурсы для каждого мероприятия вмешательства и его итоги, а также определили результаты каждой составляющей и эффект вмешательства. Также с помощью нисходящего анализа были определены затраты, связанные с вмешательством.

**Результаты:**

Охват вмешательства составил 60% целевого населения (4049 из 6780 человек), причем на каждого человека, прошедшего процедуру скринингового обследования, было затрачено 17 долларов США. Доступ к лечению получили 68% (660 из 976) человек, которым оно было назначено, и затраты на каждого больного гипертонией, посетившего поликлинику, составили 123 доллара США. Из 660 человек, посетивших поликлинику, 27% (178) оставались в сфере оказания помощи, и затраты на каждого такого пациента составили 194 доллара США, из них у 33% (58 из 178) удалось контролировать уровень кровяного давления. Суммарные затраты на вмешательство для каждого пациента, чей уровень кровяного давления удалось контролировать, составили 3205 долларов США.

**Вывод:**

Умеренные затраты на проведение вмешательства позволили достичь уровней осведомленности о гипертонии и ее лечения, сопоставимых с уровнями, которые были достигнуты в регионах с высоким уровнем доходов. Однако удержание пациентов в сфере оказания помощи и контроль кровяного давления оказались затруднительными в условиях трущоб. Для пациентов препятствием к удержанию в сфере оказания помощи стала стоимость, нехватка времени или забывчивость.

## Introduction

Cardiovascular diseases are the leading cause of death globally, killing 17.5 million people per year and 80% of deaths from these diseases occur in low- and middle-income countries.^1^^,^^2^ Evidence suggests that the main drivers of the global cardiovascular disease epidemic are urbanization and industrialization, which lead to an increase in sedentary lifestyles, unhealthy dietary patterns, tobacco consumption and increased alcohol consumption.^3^ Hypertension is a leading risk factor for cardiovascular diseases, and its prevalence is increasing worldwide – from 25% in 2000 to a projected 40% in 2025.^4^ The rising burden of hypertension in low- and middle-income countries is amplified by the public’s low levels of awareness, treatment and control of this condition, particularly among slum residents, who typically constitute a large portion of neglected urban populations in such settings.^5^^,^^6^ Studies in slum populations suggest that when people are made aware of having hypertension they do tend to seek care.^5^^,^^6^ However, the level of adherence to treatment for hypertension remains low for several reasons, including, but not limited to, the high costs of treatment and to patients’ perceptions of a low risk of cardiovascular diseases and belief in a one-time cure for disease rather than to lifelong preventive treatment and monitoring.^7^^–^^12^

In response to the rising burden of cardiovascular disease risk factors in slum populations in Kenya,^5^^,^^6^ a community-based intervention was developed and implemented in the capital city, Nairobi. This intervention, known as SCALE UP (the sustainable model for cardiovascular health by adjusting lifestyle and treatment with economic perspective in settings of urban poverty), has been described in detail elsewhere.^13^ The intervention had multiple components with the overall aim of reducing cardiovascular diseases risk through awareness campaigns, improvements in access to screening and standardized clinical management of hypertension. The aim of this paper is to share experiences of implementing a comprehensive intervention for primary prevention of hypertension in a slum setting and to examine the processes, outcomes and costs of the intervention. The lessons learnt from this paper will be useful to policy-makers and other stakeholders looking to implement similar interventions in highly resource-constrained settings. 

## Methods

### Context

Korogocho slum, located in Nairobi, is home to about 35 000 people resident across seven villages. Within this slum, two primary health-care centres were invited to participate: a private nonprofit facility and a community-owned facility. The intervention team set up cardiovascular diseases’ clinics at the facilities, provided basic screening equipment (such as blood pressure monitors), and trained a pair of nurses and clinical officers in each clinic to manage patients found to have hypertension, using a standardized treatment guideline developed by the study team in line with international practice.^14^ Although most patients made out-of-pocket payments for services received, the clinics offered services at highly subsidized prices, which were possible through donor funding. It was not practical in this setting to implement an intervention for all cardiovascular risk factors and therefore treatment focused on blood pressure screening and prescription of anti-hypertensive medication: hydrochlorothiazide, nifedipin or enalapril. If indicated, patients who also had diabetes were placed on metformin.

### Study design

The intervention itself was part of a quasi-experimental study. For this analysis we aimed to measure the outcome and impact of each stage of the intervention in terms of hypertension control. The intervention study was approved by the Kenya Medical Research Institute’s national ethics review committee (NON-SSC protocol no. 339; current controlled trials no. ISRCTN84424579). All participants gave written informed consent to participate in the intervention both at the household screening and at the clinics.

### Intervention

The intervention was developed based on a modelling exercise, described in more detail elsewhere.^13^^,^^15^ The intervention was implemented for 18 months from August 2012 and had four components: (i) raising awareness about cardiovascular diseases; (ii) improving access to screening; (iii) facilitating access to treatment; and (iv) promoting long-term retention in care.

A total of 50 community health workers were recruited and trained to conduct door-to-door household visits to raise awareness about the burden of cardiovascular diseases in the community and provide information about opportunities for screening, to conduct the screening and to provide brief counselling among the eligible population. The community health workers were trained for seven days at the implementing institution’s offices, with teaching facilitated by the project’s senior researchers in a classroom setting and including practice sessions. Health workers each received US$ 6 per day for transportation reimbursement.

Eligible people were all adults aged 35 years or older resident in Korogocho slum area who were listed in the database of the Nairobi urban health and demographic surveillance system^16^^,^^17^ and who consented to participate. Anthropometric and clinical measurements were taken at participants’ homes, including height, weight, waist and hip circumference, blood pressure and blood glucose (early morning, dried blood-spot testing). Community sensitization about the household visits was conducted via local radio campaigns, community meetings and religious gatherings.

All persons with elevated blood pressure (≥ 140 mmHg systolic and/or ≥ 90 mmHg diastolic)^18^ were referred by the community health worker to either of the two participating clinics. As an incentive for patients to seek care, community health workers gave each referred person a paper voucher that entitled him or her to receive a free 1-month supply of medication – valued at about United States dollars (US$) 1.8 – on their first clinic visit. All subsequent monthly medication prescribed at the clinic, however, was to be paid for by the patients. The consultation and laboratory tests were provided free of charge at these clinics, as usual in public primary health-care facilities in Kenya. To motivate them to follow-up each patient, community health workers received an incentive of US$ 3.0 per appropriately referred patient who visited the health facility.

To promote retention in care, patients receiving treatment were organized into support groups by village. Each support group received an incentive – a group reduction in the price of medication by one-third (approximately US$ 0.6) – if they collectively achieved 80% or more attendance to follow-up visits for a consecutive period of six months. Financial incentives were also offered to community health workers to organize the support groups: US$ 1.8 per support group participant attending the clinics for at least six consecutive months as scheduled. Finally, we sent monthly mobile phone short message service (SMS) reminders to patients reminding them of scheduled clinic appointments.

### Data collection and analysis

#### Data sources

The main sources of data were administrative records, activity reports, minutes of meetings and other relevant records. However, we supplemented these data with data sourced from population- and clinic-level surveys conducted at baseline and at the end of the intervention period.^19^ The population-level survey was conducted with randomly sampled participants in the study community at baseline (August to December 2012) and endline (February to April 2014). The clinic survey involved structured interviews with patients attending the clinics only. Data for the cost analysis were collected from financial records and time sheets and interviews with staff.

#### Processes and outcomes

To describe the processes we first listed the activities involved in the intervention and the inputs needed to implement each activity: for example, provision of facilities (input) required for training of community health workers (activity). We then described the result of each activity: for example, the number of community health workers trained (output).

To evaluate the outcomes of the components of the intervention we determined the number of people participating at each stage of the intervention and calculated the following measures: the proportion of the target population who were screened and referred to the clinic for treatment (awareness-raising and screening); the proportion of people with high blood pressure referred who attended the clinic for treatment (access to treatment); and the proportion of people who attended for treatment and made six or more clinic visits within a 12-month period (retention in care). We sought to identify possible explanations for the outcomes observed in each stage of the continuum of care. For screening and awareness we used field reports to document the reasons why not every prioritized adult was reached by the intervention. For treatment-seeking and retention in care, we conducted semi-structured interviews with a random sub-sample of referred patients who defaulted from scheduled visits or never attended the clinics.

#### Impact

To evaluate the overall impact of the intervention we collected data from anonymized routine medical records from the two clinics and calculated the levels of blood pressure control achieved among patients during the intervention. The main impact measure was the percentage of all patients retained in care (defined as patients with six or more clinic visits within a 12-month period) whose blood pressure was controlled to below 140/90 mmHg. We also calculated the percentage change in the mean systolic and diastolic blood pressures of these patients between their first and sixth clinic visits.

#### Costs

The costs of the intervention were estimated from a provider perspective and expressed in US$, based on the average conversion rate in 2013 of Kenyan shillings 85 to US$ 1. We included all service and above-service level costs for each itemized activity per intervention component for the 18-month duration of the intervention. We excluded evaluation and research-related activities as they are not a part of service delivery. We used a top-down costing approach by allocating costs to each component of the intervention, then to activities by input type (Box 1). This was done through a review of financial records and time sheets and interviews with staff. Part of the management staff costs were first allocated out proportionally to the time spent on other projects. This was ascertained through interviews. The intervention staff costs were then allocated to implementation activities based on the relative duration of research and implementation activities.

Box 1 **Costs considered in each input category of the 18-month community-based intervention for hypertension management in Kenya, 2012–2013**

**Personnel input:**

Salaries of all categories of staff and consultants

**Commodities and supplies input:**

Costs of drugs, tests, consumables and all training and communication materials

**Training input:**

Costs of space, travel, food and accommodation for participants, excluding staff costs

**Capital cost input:**

Costs of equipment, furniture, buildings (tents, floors) and labour to set up

**Building operating and maintenance input:**

Costs of communication, security, cleaning and repairs

**Transport input:**

Mileage allowance for supervision visits

**Intervention activities input:**

All payments for incentives to community health workers and patients, short message service reminders, community mobilization and adherence support

**Indirect expenses input:**

Reported overhead expenses

For all costs, we first allocated those costs that could be clearly tracked to a particular component. For the remaining shared costs, we allocated them across components based on the level of effort in hours dedicated to the activities in each component. We accounted for additional costs such as security escorts for our staff due to the field conditions. We report economic costs including items for which there were no financial transactions, for example rental of clinic space (these were valued using market prices). Capital costs were converted to an annual rate using a discount rate of 3%.

The costs for all inputs by itemized activities within each intervention component were then totalled to determine the total amount spent on that component. We then divided the total cost per component by the quantifiable unit of outcome per component, resulting in the cost per unit of outcome per intervention component. Finally, we totalled the cost of all components and divided that by the number of people with blood pressure under control to obtain the cost per unit of health gain (patient with blood pressure controlled and retained in care).

## Results

### Processes and outcomes

Table 1 shows the details of all the activities and inputs and the resultant outputs for each component of the intervention.

**Table 1:** Results of process evaluation of the community-based intervention for hypertension management in Kenya, 2012–2013

Intervention component by input category	Inputs	Activities	Outputs
**Awareness and screening**			
Community gatherings *(baraazas)*	Banners, public address system, facilitators (community leaders, expert patients)	7 *baraazas* held	Estimated between 50 and 80 people attended each meeting
Religious services	Facilitators (community health workers, religious leaders)	21 religious meetings held	Estimated between 30 and 50 people attended
Radio jingle	Jingle content developer, local radio station (Koch FM)	1 jingle lasting 50 seconds aired 3 times daily for 3 weeks	Koch FM radio listener numbers estimated at 250 000 people
Community health workers	Facilitators (medical/research officers), training facilities, allowances	1 training and 1 refresher training held	50 community health workers trained^a^
Door-to-door screening	Community health worker allowances, screening equipment and materials	39 community health workers conducted door-to-door screenings	4049 people screened
Referral	Free vouchers, confirmation of blood pressure by supervisor	39 community health workers conducted referrals	976 people referred
**Treatment**			
Clinic staff	Facilitators (medical/research officers), training facilities, allowances	1 training and 1 refresher training held	2 nurses, 2 clinical officers and 1 medical records clerk trained
Standard treatment guidelines	Meetings and review by stakeholders	1 main meeting held with stakeholder. Guideline reviewed mostly by email correspondence	1 guideline document published
Upgrading and equipping of clinics	Construction of consultation area, equipment	2 clinics upgraded. Concrete floor constructed and tent erected in 1 clinic. Both clinics received 2 sets of screening equipment and light furniture for consulting areas	2 clinics upgraded
Management of referred patients at clinics	Clinic staff allowances, utilities and supplies (including medication)	Clinics held twice a week for 17 months	845 people attended clinic first time, of whom 660 were eligible for recruitment into care
**Retention in care**			
Follow-up of defaulters	Community health workers’ allowances (including incentives) and resources	188 defaulters followed up and interviewed by community health workers	46 defaulters returned to clinic after follow-up
SMS reminders	Bulk SMS application	4519 SMS reminders sent	660 patients received SMS reminders
Support groups	Community health workers, facilitators, incentives	7 support groups formed and 28 support groups held	371 people attended support groups

SMS: short message service.

^a^ Although 50 community health workers were trained, not all were deployed to conduct screening. Some dropped out of the study to pursue other interests.

**Fig. 1:**
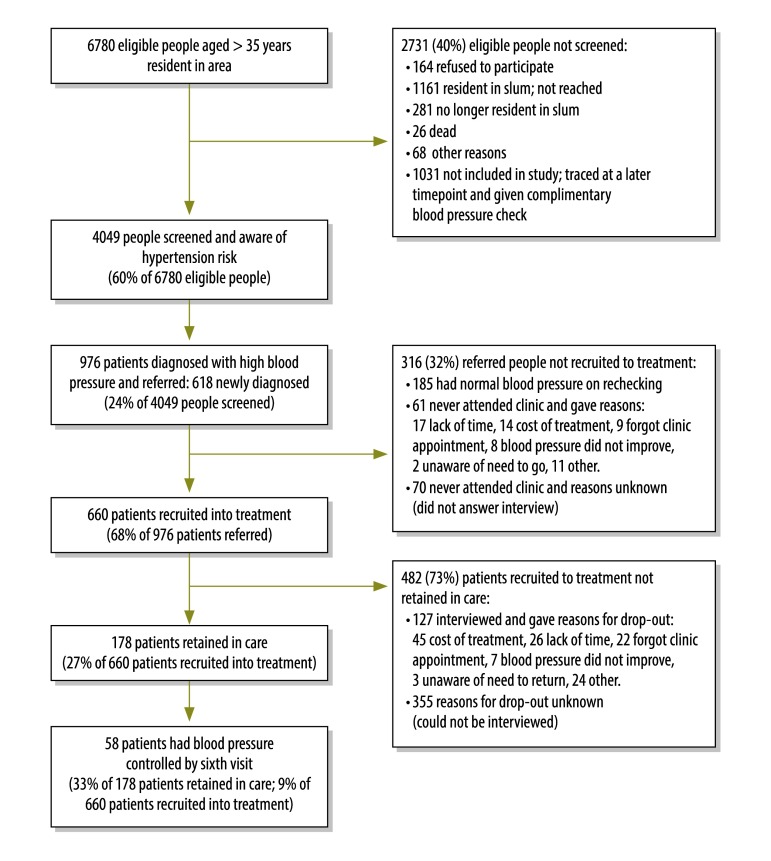
Outcome and impact indicators for each stage in the cascade of hypertension diagnosis and management in the community-based intervention in Kenya, 2012–2013

Fig. 1 summarizes the outcomes of each stage of the intervention. Community health workers successfully screened and counselled 4049 out of 6780 (60%) of the target population. The principal reasons for exclusion from the study were because the person refused to participate in the study (164; 2%), was believed to be resident in the slum but could not be reached (1161/6780; 17%), was no longer resident in the slum (281; 4%) or had died (26; < 1%). Other reasons accounted for 68 (1%) of drop-outs. A further 1031 people were not reached during the screening campaign but were traced by community health workers during the intervention period and given a complementary blood pressure check. However, we did not collect any data from these people nor did we follow them up at the clinic. Out of the 4049 people screened, 976 (24%) people with raised blood pressure were identified and referred; 358 (9%) were known to have hypertension and 618 (15%) were newly diagnosed.

Out of the 976 persons referred to the clinics, 845 (87%) attended the cardiovascular diseases’ clinic at least once. Of these, 185 were found to have normal blood pressure levels after confirmatory measurement by the nurses. Therefore 660 patients out of 976 referred (68%) started on treatment with a prescription from the clinic. Out of 131 patients who were referred to a clinic but did not attend, 61 answered the follow-up interviews conducted at their homes. The leading reported reasons for non-attendance were lack of time (17 patients; 28%), cost of treatment (14; 23%) and forgot clinic appointment (9; 15%).

By the end of the intervention period, a total of 4519 SMS messages had been sent to all patients, and seven support groups had been formed with a total of 371 persons attending the meetings at least once. The average number of support group meetings attended by each person was 3.5 over the entire intervention period.

A total of 178 out of 660 patients (27%) attending the clinics were retained in care. Out of the 482 patients not retained in care, the community health workers followed up 127 at their homes; the remainder could not be reached after up to three revisits. The main reasons for not being retained in care included cost of treatment (45 patients; 35%), lack of time (26; 21%) and forgot clinic appointment (22; 17%).

### Impact

Out of 178 patients retained in care, 58 (33%) had their blood pressure controlled by the sixth visit. This amounts to 9% of all 660 patients recruited into the clinics (Fig. 1).

The mean systolic blood pressure of those retained in care and with complete data (*n* = 177) was 161.6 mmHg at the first visit and this was reduced by 19.8 mmHg (95% confidence interval, CI: 16.0–23.6) by the sixth visit. Their mean diastolic blood pressure was 100.5 mmHg at the first visit and this was reduced by 10.4 mmHg (95% CI: 8.2–12.7) by the sixth visit. 

### Costs

Table 2 shows the total costs of the intervention by input category and intervention component. The awareness and screening component of the intervention accounted for 38% (US$ 70 071) of the total cost of US$ 185 861, access to treatment for 44% (US$ 81 337) and retention in care for 19% (US$ 34 453). Personnel was the highest input cost at 53% (US$ 99 119) of the total cost.

**Table 2:** Total costs by input category and component of the 18-month community-based intervention for hypertension management in Kenya, 2012–2013

Input category	Cost, US$			
	Awareness and screening	Treatment	Retention in care	All components (%)^a^
**Personnel**				
CHWs facilitation fee (support groups)	–	–	329	329
Programme management	9 146	32 926	21 951	64 022
Field supervisor	3 747	3 747	3 747	11 241
Field team leaders	7 169	6 452	418	14 040
Clinical staff for cardiovascular diseases’ clinics	–	9 487	–	9 487
Total	–	–	–	99 119 (53)
**Commodities and supplies**				
Medical consumables	2 042	1 602	–	3 645
Non-medical supplies	3 642	6 590	–	10 231
Medications^b^	0	0	0	0
Total	–	–	–	13 876 (8)
**Training**				
Training sessions	4 498	1 519	748	6 765 (4)
**Capital cost**				
Clinic upgrading	–	1 190	–	1 190
Equipment	19 126	1 460	–	20 586
Furniture	–	593	–	593
Total	–	–	–	22 369 (12)
**Building operating and maintenance**				
Repairs	–	12	–	12
Field communication	302	272	18	591
Field security	590	–	–	590
Cleaning	–	104	–	104
Building rent^c^	–	141	–	141
Total	–	–	–	1 437 (1)
**Transport**				
Transport for supervision visits	889	1 671	108	2 668 (1)
**Intervention activities**				
CHWs for screening and referral	7 249	–	–	7 249
CHWs for retention in care	–	–	935	935
First free treatment voucher	–	1 165	–	1 165
Community gatherings *(baraazas)*	589	–	–	589
Religious services	271	–	–	271
Radio jingle	124	–	–	124
Running of support groups	–	–	376	376
Training the trainers sessions	–	–	515	515
SMS reminders	–	–	53	53
Total	–	–	–	11 275 (6)
**Indirect expenses**				
Programme overheads (estimated at 18%)	10 689	12 407	5 256	28 352 (15)
**All categories**	70 071	81 337	34 453	185 861 (100)

CHWs: community health workers; SMS: short message service; US$: United States dollars.

^a^ Total cost of input category as a percentage of total intervention cost (US$ 185 861).

^b^ Drug costs were paid by patients.

^c^ Donated; value was estimated and included utilities (electricity, water, medical waste disposal etc.).

Notes: The average conversion rate during 2013 was 85 Kenyan shillings to US$ 1. Dashes indicate data not applicable.

Table 3 summarizes the unit costs per patient with blood pressure controlled for the three components of the intervention. The unit cost per person reached via screening and awareness was US$ 17. For access to treatment, the cost was estimated at US$ 123 per person seeking treatment. It cost US$ 194 per person to retain a patient in care. The overall cost of getting a person screened, treated, retained in care and to have their blood pressure under control was US$ 3205.

**Table 3:** Summary of costs per unit of outcome at each stage of the community-based intervention for hypertension management in Kenya, 2012–2013

Intervention component	Cost, US$	No. of people reached	Cost per person reached, US$
Awareness and screening	70 071	4 049	17
Treatment	81 337	660	123
Retention in care	34 453	178	194
Blood pressure control	185 861	58	3 205

US$: United States dollars.

Note: The average conversion rate during 2013 was 85 Kenyan shillings to US$ 1.

## Discussion

In summary, the intervention reached 60% of the target population, provided access to treatment to 68% of eligible patients with hypertension, retained 27% in care and achieved blood pressure control among 33% of patients retained in care.

These results show that, despite the intervention, the so-called rule of halves – “half the hypertensive population is undetected, half of those detected are untreated, and in half of those treated hypertension is not controlled”^20^ – applied in our setting. Studies in developing countries have shown that the levels of awareness, treatment and control of hypertension are still quite low, with control rates ranging from 4% to 47% among patients aged 35–49 years.^21^ Even worldwide, only 13% of people with hypertension have adequate blood pressure control.^22^ Our study showed that with a comprehensive community-based intervention it is possible to achieve awareness and initial treatment rates above 50%. Achieving awareness and access to treatment levels that are comparable to high-income countries is commendable, especially in unstable populations such as those in slum areas, where annual migration rates alone could reach 30%.^23^ However, retention in care and blood pressure control rates in our population remains suboptimal despite the intervention components designed to address them.

We also found that our financial incentives were not strong enough to keep the majority of patients retained in care. We believe that this finding is specific to our setting: over 90% of slum residents in Kenya make out-of-pocket payments for health^24^ and more than 50% report being food insecure.^25^ Moreover, many of those who defaulted from the clinic cited cost as the main reason. In other words, although treatment costs were subsidized, it was still a barrier to care. Cost is an issue in other settings too. A study in 36 mostly low- and middle-income countries found that 1 month of daily treatment with one hypertensive drug cost on average 1.8 days’ wages.^26^ The World Health Organization has set a global target of a 25% reduction in the prevalence of hypertension by 2025.^27^ If this target is to be achieved, then mechanisms need to be found to make drugs more affordable, as has been achieved with tuberculosis treatment and antiretroviral therapy (ART) to supress human immunodeficiency virus (HIV) infection.

The cost of the entire intervention per person with blood pressure controlled was US$ 3205. This compares favourably to other multifaceted public health interventions in Kenya. For example, the implementation of the option B+ approach, in which HIV-positive pregnant women are started immediately on ART and continued for life – a comparable intervention as it includes screening, diagnosis and chronic treatment – was recently estimated to be US$ 6015 per infection averted.^28^ However, when we place the cost of each component of the hypertension intervention in the context of other public health interventions for cardiovascular disease prevention we find that our costs are high. For example, a recent systematic review found that the costs of using general medical practitioners in community hypertension programmes ranged from only US$ 0.81 to US$ 8.67 per patient per year, versus our unit cost of US$ 123 per person seeking treatment.^29^ Furthermore, the per capita cost of the intervention was almost three times higher than the gross domestic product per capita of Kenya in 2014 (US$ 1290).^30^ Yet compared with the treatment costs of other chronic diseases our costs are favourable. For example, from a provider perspective it costs US$ 273 and US$ 258 to treat drug-susceptible tuberculosis in lower middle-income and low-income countries, respectively.^31^ The median cost of antiretroviral therapy per patient per year is estimated to range from US$ 682 to US$ 1089 in low-income countries and from US$ 156 to US$ 3904 in lower middle-income countries.^32^

The study has several limitations. First, due to budgetary limitations we were unable to collect all the data to compute the cost–effectiveness of our intervention, as originally intended in our study protocol for a quasi-experimental community-based trial.^13^ This meant that we did not obtain fasting blood glucose levels for all study participants to determine their 10-year cardiovascular diseases risk.^14^ Second, our study was conducted in a slum setting, which limits the generalizability of our findings to other settings. Nonetheless, as slums are said to constitute up to 60% of urban areas in low- and middle-income countries^33^ we believe our findings will be useful to urban health practitioners in other settings.

In conclusion, the intervention achieved reasonable success in terms of raising awareness and hypertension treatment levels in a challenging resource-constrained setting at a cost that in principle would be regarded as affordable when compared with public health interventions such as ART. We recommend, however, that further research be conducted to address low levels of retention in care and of blood pressure control in such settings. In terms of scalability and sustainability, we believe that the strength of the intervention is that it simplifies the process of identifying persons with high blood pressure at community level and linking them into care. This characteristic has the potential to make it applicable to other contexts. Indeed, certain elements of the intervention have been implemented successfully in other contexts. For example, an observational study in Bangladesh, Guatemala, Mexico and South Africa demonstrated that community health workers could do community-based screenings to predict cardiovascular disease risk as effectively as physicians or nurses.^34^ The clinics that we set up as part of this study were handed over to the local government in Kenya and continue to be operational in 2016.
